# Synthesis of Folic Acid-Functionalized Hybrid Mesoporous Silica Nanoparticles and In Vitro Evaluation on MCF-7 Breast Cancer Cells

**DOI:** 10.3390/ijms27021092

**Published:** 2026-01-22

**Authors:** Marta Slavkova, Yordan Yordanov, Christina Voycheva, Teodora Popova, Ivanka Spassova, Daniela Kovacheva, Virginia Tzankova, Borislav Tzankov

**Affiliations:** 1 Department of Pharmaceutical Technology and Biopharmaceutics, Faculty of Pharmacy, Medical University of Sofia, 1000 Sofia, Bulgaria; hvoycheva@pharmfac.mu-sofia.bg (C.V.); tpopova@pharmfac.mu-sofia.bg (T.P.); 2 Department of Pharmacology, Pharmacotherapy and Toxicology, Faculty of Pharmacy, Medical University of Sofia, 1000 Sofia, Bulgaria; yyordanov@pharmfac.mu-sofia.bg (Y.Y.); vtzankova@pharmfac.mu-sofia.bg (V.T.); 3 Institute of General and Inorganic Chemistry, Bulgarian Academy of Sciences, 1113 Sofia, Bulgaria; ispasova@svr.igic.bas.bg (I.S.); didka@svr.igic.bas.bg (D.K.)

**Keywords:** doxorubicin, breast cancer, folate targeting, lipid coating, mesoporous silica nanoparticles, hybrid nanoparticles, cardiotoxicity

## Abstract

Folate receptor alpha is expressed at low levels in normal tissues, but is elevated in aggressive breast cancer types and can be utilized for targeted nanoparticle delivery. Hence, we prepared a hybrid nanocarrier based on in-house synthesized mesoporous silica nanoparticles (MSNs) which were further lipid-coated and reinforced with folic acid (FA). Thorough physicochemical evaluation was performed including dynamic light scattering (DLS), powder x-ray diffraction (PXRD), thermogravimetric analysis (TGA), and nitrogen physisorption. In vitro dissolution of the model drug doxorubicin was carried out in release media with pH 7.4 and pH 5.5. The cytotoxic potential and cellular uptake were investigated in MCF-7 breast cancer cells via the MTT assay, doxorubicin fluorescence measurement, and microscopy. The potential amelioration of doxorubicin’s cardiotoxicity was evaluated in vitro on the H9c2 cell line. The results showed MSNs with significant pore volume (1.38 cm^3^/g) and relatively small sizes (98.05 ± 1.34 nm). The lipid coat and FA attachment improved the physicochemical stability and sustained release pattern over 24 h. MSNs were non-toxic, while when doxorubicin-loaded, they caused moderate cytotoxicity. The highest cytotoxic activity was observed with folate-functionalized, doxorubicin-loaded nanoparticles (NPs). Even though non-loaded folate-functionalized NPs exhibited significant cytotoxicity, their physical mixture with doxorubicin was inferior in MCF-7 cytotoxicity as opposed to the corresponding loaded nanocarrier. Fluorescence-based quantification showed a higher intracellular accumulation of doxorubicin when delivered via NPs. These results demonstrate the potential to use folate-functionalized NPs as carriers for doxorubicin delivery in breast cancer cells. Its cardiotoxicity was significantly reduced in the case of loading onto the folic acid-functionalized lipid-coated MSNs. All these findings provide a promising proof-of-concept, although further experimental validation, particularly regarding targeting selectivity and safety, is required.

## 1. Introduction

Breast cancer is the most commonly diagnosed cancer in women and accounts for about 30% of all cancer cases [1]. However, most therapeutic modalities have significant side effects and new, more selective therapies are actively researched [2]. Folate receptor alpha (FRα) plays a significant role in breast cancer, particularly in aggressive subtypes such as triple-negative breast cancer (TNBC), in which it is overexpressed in approximately 71% of the cases [3]. FRα facilitates receptor-mediated endocytosis, which enables the efficient internalization of folate-conjugated nanoparticles into cells, which express it. This property can enhance the intracellular delivery of therapeutic agents, improving their efficacy [4,5]. Being a small-molecule, nonimmunogenic compound with practically negligible influence on the pharmacokinetic behavior, it is an excellent candidate for the surface functionalization of nanoparticles with active targeting [6,7]. 

The study of nanoparticles (NPs) in the diagnosis and therapy of cancer has been a subject of high scientific interest. Nanocarriers provide numerous opportunities to improve drug solubility, modify drug release patterns, and improve drug stability [8]. Furthermore, the high payload and possibility to passively or actively target tumor cells allow for limited healthy tissue exposure and hence decreases in the off-site toxicity [8,9]. The passive aiming at cancerous cells relies on the so-called EPR (enhanced permeation and retention) effect. It is a selective penetration into tumors due to the extensive angiogenesis and the associated high permeation of compounds. The EPR effect is dependent on the nanoparticle’s physicochemical properties such as size, shape, and surface properties and the optimal values depend on the tumor and nanoparticle type and are associated with each other [10]. On the other hand, active transport and retention (ATR) is governed by active transport such as endocytosis, vesiculo-vacuolar organelles, and/or immune cell migration [11]. Active targeting is dependent on specific ligands, such as small molecules, carbohydrates, peptides, and antibodies, which have high affinity and specificity for selective tumor cells [12]. Combining both strategies of passive and active drug targeting through a nanotechnological approach offers a vast horizon of chemotherapeutic options with limited adverse effects. Doxorubicin is an example of a potent chemotherapeutic effective in many solid tumors but simultaneously its major drawback is the inherent cardiotoxicity [13]. Therefore, it is not surprising that many nanotechnological approaches have been investigated in dealing with the improvement of its efficacy and safety profile [14]. Hence, in the present work we selected doxorubicin (Dox) as a model drug.

There are a wide range of investigated nanocarriers including inorganic, lipid, polymeric nanoparticles, dendrimers, and others. Each of them is characterized by some advantages and disadvantages, hence the appearance of hybrid nanocarriers which can combine the attributes of at least two distinct classes of nanosized drug delivery systems. Mesoporous silica nanoparticles (MSNs) and lipid-based nanocarriers have proven themselves as one of the most attractive and advantageous ones. The MSNs are generally regarded as safe (GRAS) by the American Food and Drug Administration [15]. Their unique physicochemical properties such as high specific surface area and variable pore size, together with the possibility for surface functionalization, allow the loading of hydrophobic and hydrophilic molecules with significant capacities [16]. On the other hand, lipid nanoparticles have proven to be clinically applicable since the marketed nano-products or the ones in clinical processes based on the lipid platform occupy 33% of all nanoparticles [12]. Even though there are a variety of lipid-based carriers, they all possess high biocompatibility and biodegradability, high stability, and a programmable surface chemistry. By combining both types of nanocarriers, it is possible to optimize many parameters together. There are several studies that prove that this approach could increase circulation time, improve colloidal stability, and allow the incorporation of hydrophilic and hydrophobic drugs simultaneously, as well as modify the drug release [17,18,19]. The lipid can be applied as a surface coat on the inorganic core. The coating can be in the form of either a supported or hybrid layer depending on the method of its preparation [20]. In some cases, lipids can self-assemble in the presence of an aqueous surfactant solution and could be layered as a supported coat. The non-lamellar structure resembles almost completely the human cells and is therefore related with improved intracellular drug delivery [21]. Further surface functionalization with targeting ligands additionally can improve the therapeutic potential of these hybrid nanoparticles. Folic acid functionalization is a promising strategy for selective delivery to cancer subtypes overexpressing FRα [22].

Based on this conceptualization, in this work, we synthesized the mesoporous silica nanoparticle core which was subsequently lipid-coated and folic acid was chemically attached to its surface. The research aimed at obtaining a hybrid inorganic–lipid nanocarrier with the potential to increase the doxorubicin cytotoxicity to breast cancer cells (MCF-7) and therefore to improve the antiproliferative drug’s efficacy in vitro. In addition, we hypothesized that Dox loading in a hybrid lipid and folic acid-coated mesoporous silica nanoparticle (MSNs-LFA-Dox) would reduce the cardiotoxicity of Dox in human H9c2 cardiomyocyte cells.

## 2. Results

**Figure 1:**
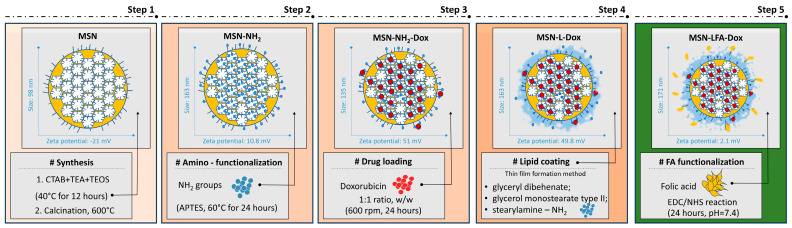
Preparation scheme of folic acid-functionalized hybrid mesoporous silica nanoparticles. The following abbreviations are used: MSN—mesoporous silica nanoparticle; MSN-NH_2_—amino-functionalized MSN; MSN-NH_2_-Dox—doxorubicin-loaded MSN-NH_2_; MSN-L-Dox—lipid-coated MSN-NH_2_-Dox; MSN-LFA-Dox—folic acid (FA)-functionalized hybrid nanoparticle loaded with doxorubicin; CTAB—cetyltrimethylammonium bromide; TEA—triethanolamine; TEOS—tetraethyl orthosilicate; APTES—aminopropyltriethoxysilane; EDC—N-ethyl-N′-(3-(dimethylamino)-propyl) carbodiimide; NHS—N-hydroxysuccinimide.

In this work, we report the successful synthesis, lipid coating, and folic acid functionalization for improving the doxorubicin delivery to cancerous cells. The proposed preparation scheme step by step with some key findings is presented in Figure 1.

### 2.1. Dynamic Light Scattering and Transmission Electron Microscopy Analysis

The preparation method resulted in relatively small nanoparticles with negative zeta potential (Table 1). The functionalization, drug loading, and lipid coating are associated in general with an increase in the hydrodynamic diameter which is correspondent to the surface modification. The change in the zeta potential (Table 1) is also a proof for each successful step of preparation

The amino-functionalized nanoparticles were subsequently loaded with doxorubicin. The corresponding lipid-coated and FA-functionalized nanocarriers are based on the plain MSN-NH_2_ and doxorubicin-loaded sample MSN-NH_2_-Dox, respectively. The determination of the encapsulation efficiency for the MSN-NH_2_-Dox revealed 28.76 ± 2.16%. The loading capacity was 14.38 ± 1.53%. The MSN-NH_2_-Dox samples were utilized in the subsequent lipid coating and folic acid functionalization. Hence, the loading capacity was reduced to 5.78 ± 0.64% due to the increase in the weight of the nanocarrier.

**Figure 2:**
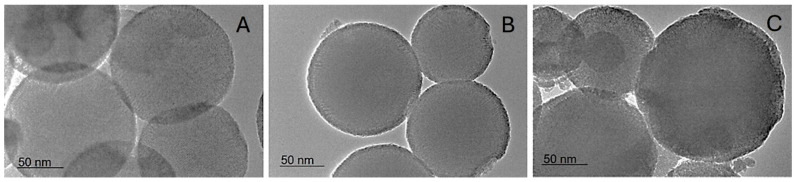
TEM images unmodified MSN carrier (**A**), lipid-modified variant—MSN-LFA (**B**), as well as the doxorubicin-loaded sample MSN-LFA-Dox (**C**).

Similar sizes are observed by the TEM investigation. Additionally, spherical shapes and mesopores can be seen (Figure 2).

### 2.2. Nitrogen Physisorption

**Figure 3:**
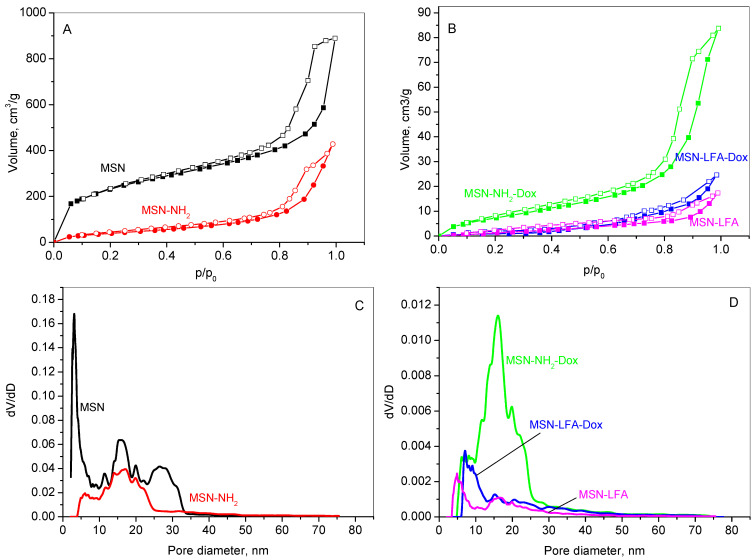
Adsorption/desorption isotherms of MSN, MSN-NH_2_ (**A**), MSN-LFA, MSN-NH2-Dox, MSN-LFA-Dox (**B**), PSD of MSN, MSN-NH_2_ (**C**), and PSD of MSN-LFA, MSN-NH2-Dox, MSN-LFA-Dox (**D**).

The textural properties of the unmodified MSN carrier, its aminated form (MSN-NH_2_), the lipid and folic acid-modified variant (MSN-LFA), as well as the doxorubicin-loaded samples (MSN-NH_2_-Dox and MSN-LFA-Dox), were analyzed using nitrogen physisorption. The adsorption–desorption isotherms are shown in Figure 3A,B, while the corresponding pore size distributions (PSDs) are presented in Figure 3C,D. The textural parameters derived from these isotherms are summarized in Table 2.

The results reveal that all samples show type IV isotherm which is typical for mesoporous materials according to the IUPAC classification [23]. MSN, MSN-NH_2_ (Figure 3A), and MSN-NH_2_-Dox (Figure 3B) exhibit mixed H1–H4 type hysteresis loops, characteristic of mesoporous materials with a complex pore size distribution. It suggests the coexistence of various pore shapes and dimensions within the material, including both cylindrical and slit-like pores, as well as others with distinct features. This points to a complex and potentially heterogeneous pore architecture, which can significantly affect the material’s surface area, pore size distribution, and overall performance. The hysteresis loops for the lipid-modified MSN-LFA and MSN-LFA-Dox change slightly in form, revealing some textural changes in the samples. The pore size distribution presents a multimodal curve of MSNs in a range of 2–30 nm, with a mean pore diameter of 3 nm. The amination of MSNs leads to a narrower pore distribution (Figure 3C); however, the filling of the narrower pores is visible, leading to a larger average pore size. A reduced specific surface area due to functionalization is registered. Lipid modification affects the organization of the pores within the silica structure. The lines in Figure 3D show that the lipid chains lining the pores narrow the effective diameters of the lipids-containing samples, shifting the pore size distribution curves to lower pore diameters. The specific surface area of the doxorubicin-loaded sample slightly increases due to the amorphisation of the drug onto the aminated mesoporous silica nanoparticles.

### 2.3. Thermogravimetric Analysis

**Figure 4:**
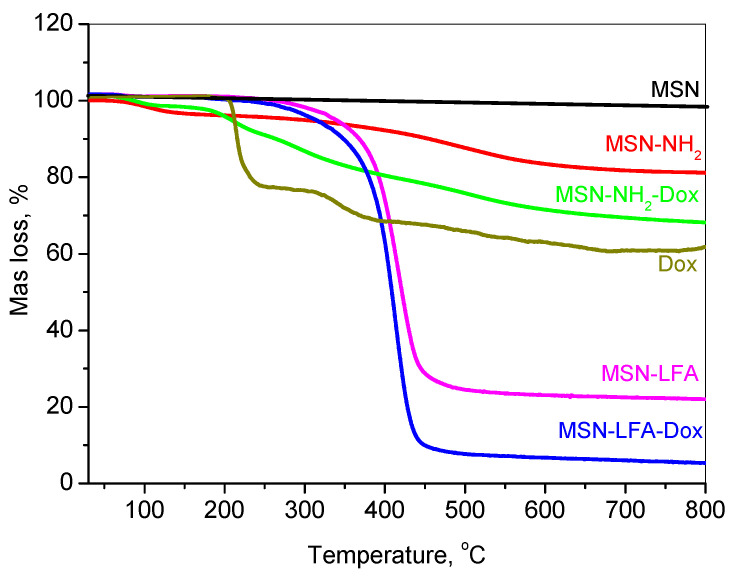
Thermogravimetric curves of MSN, MSN-NH_2_, MSN-NH_2_-Dox, Dox, MSN-LFA, and MSN-LFA-Dox.

The thermal decomposition profiles of doxorubicin-loaded and functionalized mesoporous silica nanoparticles are presented in Figure 4.

The thermogravimetric analysis (TGA) of free doxorubicin (Dox) revealed no initial mass loss attributable to the solvent or water, indicating its anhydrous nature. Between 150 and 250 °C, a mass loss of approximately 20% was observed, corresponding to the thermal decomposition of labile functional groups, such as the glycosidic moiety. Further degradation occurring in the 250–380 °C range, resulting in an additional ~17% mass loss, is indicative of the breakdown of aromatic ring systems and the formation of carbonaceous residues. Continued mass loss up to 800 °C is associated with the gradual degradation of these residues into gaseous products, ultimately leaving behind a carbonaceous residue.

The mesoporous silica nanoparticle (MSN) framework exhibited high thermal stability under an inert argon atmosphere. Negligible mass loss (~2%) was recorded up to 800 °C, attributable only to minor dehydroxylation processes, with no observable degradation of the silica matrix.

For amino-functionalized MSNs (MSN-NH_2_), a mass loss of ~3% occurred below 150 °C due to the desorption of physically adsorbed water. In the 150–550 °C range, approximately 12% of the mass was lost due to the thermal decomposition of grafted amine groups. An additional ~3% loss, attributed to silica network densification, resulted in a total mass loss of 18%, from which the amine functional group content was estimated at ~16%.

The thermogram of MSN-NH_2_-Dox displayed a composite thermal behavior representative of both MSN-NH_2_ and Dox. The total mass loss reached 32%, indicating a Dox loading content of approximately 14%.

Lipid and folic acid-modified MSN samples (MSN-LFA and MSN-LFA-Dox) exhibited similar thermal degradation profiles, differing primarily in the extent of mass loss due to the presence of Dox. For MSN-LFA, the total mass loss was 78%, while for MSN-LFA-Dox, it increased to 93%. Based on comparative analysis with MSN-NH_2_ and MSN-LFA, the lipid content was estimated to be approximately 60%.

### 2.4. Powder X-Ray Diffraction

**Figure 5:**
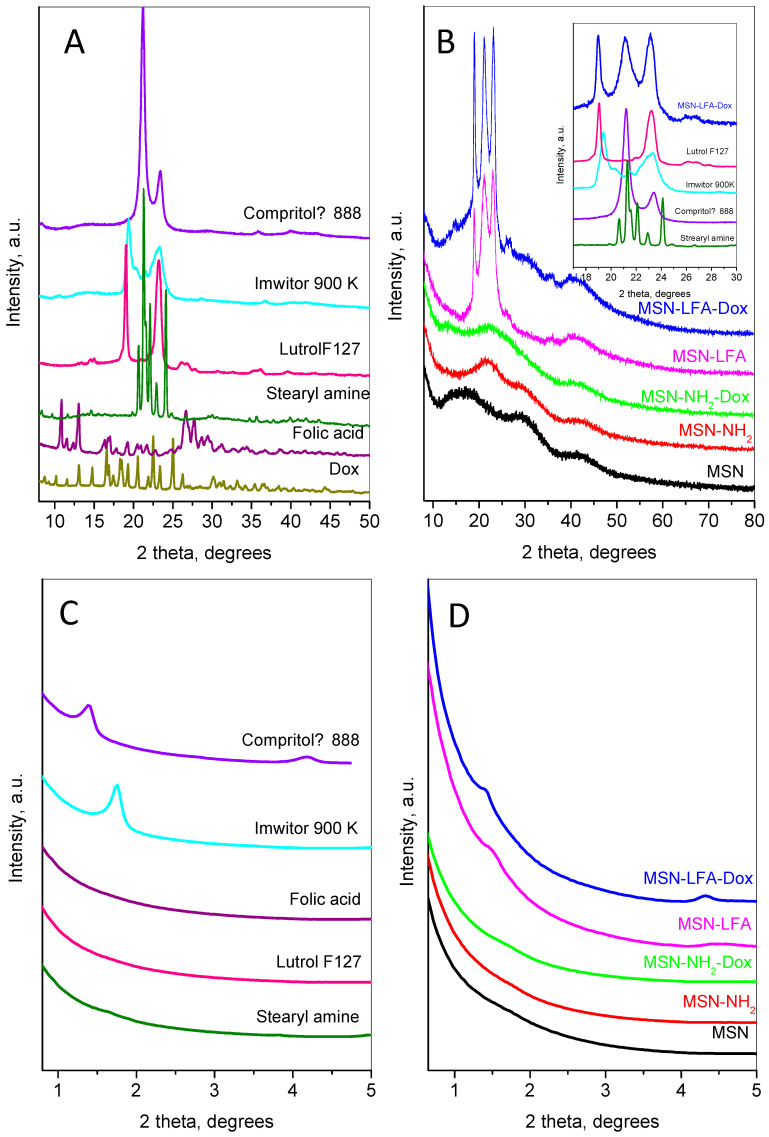
Wide-angle and small-angle X-ray diffraction patterns of doxorubicin, Compritol^®^ 888 ATO, Imwitor 900K, stearylamine, folic acid, and Lutrol F127 (**A**,**C**) and of MSN, MSN-NH_2_, MSN-NH_2_-Dox, MSN-LFA, MSN-LFA-Dox (**B**,**D**). The inset in (**B**) compares the characteristic part of the diffraction patterns of MSN-LFA-Dox with the corresponding region of the patterns of the compounds used for lipid modification.

The small- and wide-angle parts of powder diffraction patterns of the main compounds involved in the preparation of the nanoparticles are shown in Figure 5.

The doxorubicin’s wide-angle diffraction pattern (Figure 5A) consists of strong and narrow peaks, revealing its high crystallinity. The observed set of inter-planar distances is consistent with SG P21. The calculated unit cell parameters were a = 10.236(2) Å, b = 6.255(1) Å, c = 20.284(4) Å, and β = 96.80(2)°, close to those observed in [24]. The mean crystallite size was determined to be 76 nm. The pattern of folic acid corresponds to the reference pattern in the ICDD database PDF 2#00-065-1262. The pattern of stearylamine is indexed in the monoclinic SG P21 with unit cell parameters a = 5.690(2) Å, b = 7.357(2) Å, c = 50.96(1) Å, and β = 119.29(1). The diffraction pattern of Lutrol F127 contains two strong peaks: a narrow one at 18.95° 2θ indexed as (120) and a slightly broader complex one at 23.13° 2θ corresponding to the planes (112), (20-4), (032), and (1-3-2). Several low-intensity peaks are also presented. The unit cell is monoclinic P21/a, with parameters a = 8.019(8) Å, b = 13.09(1) Å, c (fiber axis) = 19.45(2) Å, and β = 125.03(4)°. The diffraction pattern of Imwitor^®^ 900K is identical to that already published in [25] with two strong peaks at 19.3 and 23.3° 2θ. The diffraction pattern of Compritol^®^ 888 ATO exhibits a strong peak at 21.1° 2θ and one with lower intensity at 23.4° 2θ characteristic of the orthorhombic β’form of triglycerides [26].

The low-angle part of MSN (Figure 5D) does not show any peaks related to long pore ordering, while its wide-angle pattern (Figure 5B) consists of three wide humps centered at 16.9, 29.8, and 42.6 °2θ. After amino-functionalization, the first hump becomes stronger and shifts to a higher angle (22.1 °2θ), while the other two humps remain un-shifted. The XRD pattern after the incorporation of doxorubicin on the MSN-NH_2_ indicates the amorphization of the Dox accompanied with the appearance of two additional humps at 13.7 and 26.2 °2θ.

The results of lipid coating can be traced by analyzing the diffraction pattern of MSN-LFA, Figure 5B. The wide-angle part presents three strong peaks at 18.9, 21.2, and 23.1 °2θ and two wide, low-intensity peaks at around 26.1 and 35.7 °2θ. Comparison with the individual patterns of Compritol^®^ 888 ATO, Imwitor^®^ 900K, stearylamine, folic acid, and Lutrol F127 (see the inset of Figure 5B) reveals the contribution of these components as follows: the first peak at 18.9 is a superposition of the peaks of Lutrol F127 and Imwitor^®^ 900K; the second broad peak includes lines of Compritol^®^ 888 ATO and stearylamine; the third peak is also complex and contains contributions of Lutrol F127, Imwitor^®^ 900K, and Compritol^®^ 888 ATO.

The small-angle region of the diffraction patterns provides additional information on the results of lipid coating (Figure 5C,D). The first peak of Imwitor^®^ 900K appears at 1.39 °2θ (d ≈ 63.2 Å) and the second one at 4.18 °2θ, while Compritol^®^ 888 ATO shows only one peak at 1.75 °2θ (d ≈ 50.3 Å). These peaks are connected with the interlayer distances. Stearylamine also shows peaks in this region related to the longest unit cell parameter (d ≈ 51 Å). For the MSN-LFA, the peak has an intermediate position and is broader than the corresponding peaks of Imwitor^®^ 900K and Compritol^®^ 888 ATO, suggesting that interlayer disordering may be due to the influence of the MSN surface. For the MSN-LFA-Dox, the peaks are closer to those of the Compritol^®^ 888 ATO, leading to the conclusion that Dox loading prevents strong interactions of the lipids with the MSN surface.

### 2.5. Drug Release Profile

**Figure 6:**
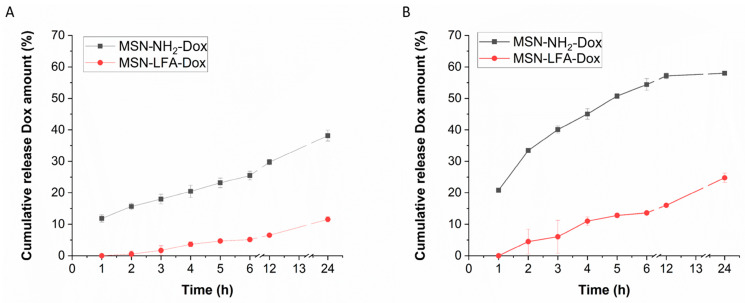
In vitro dissolution profiles of the doxorubicin-loaded nanoparticles in phosphate buffer with pH 7.4 (**A**) and 5.5 (**B**) (mean ± SD; *n* = 3).

The biopharmaceutical study of the release behavior in different pH media, namely pH 7.4 (physiological extracellular pH of healthy tissues [27]) and pH 5.5 (typical for tumor microenvironment [28]), is presented in Figure 6. An incomplete release is observed and a sustained pattern can be identified.

### 2.6. Cell Experiments

#### 2.6.1. Cell Viability Assay on MCF-7 Breast Cancer Cell Line

**Figure 7:**
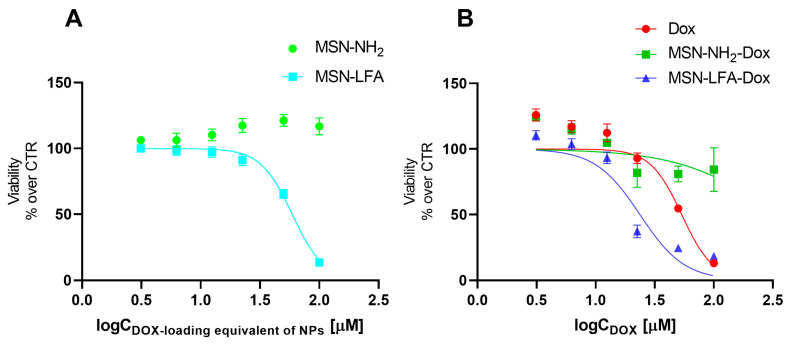
Effects of non-loaded nanoparticles MSN-NH_2_ and MSN-LFA panel (**A**) and free Dox (3.06–100 µM) and Dox-loaded MSN-NH_2_-Dox and MSN-LFA-Dox panel (**B**) on MCF-7 cell viability after 24 h treatment. In panel A, nanoparticle concentrations are expressed as Dox loading equivalent (µM) to allow direct comparison with Dox-loaded formulations. In panel B, the concentrations of loaded Dox were equimolar to those of the free drug. The results are expressed as means ± SD of triplicate assays (*n* = 3).

The effects of empty MSN-NH_2_ and MSN-LFA nanoparticles on the cell viability of MCF-7 breast cancer cells were evaluated using MTT assay (Figure 7A). For the sake of comparison, we tested the non-loaded nanoparticles in the exact same concentrations and range as in the experiments with corresponding doxorubicin-loaded nanoparticles. The hypothesis we aimed to evaluate is whether the observed cytotoxic effects of the loaded drug delivery system were due to the doxorubicin itself, or due to the cytotoxicity of the nanoparticles alone. The MTT test is a widely used and reliable method for the evaluation of the total metabolic activity of the cells, specifically reflecting on the effects on mitochondrial function. The results showed that non-functionalized MSN-NH_2_ exerted no cytotoxicity on MCF-7 cells. Interestingly, folate-functionalized MSN-LFA nanoparticles were not cytotoxic in the lower concentration range (3.06–25 µM), but they showed an increasing cytotoxicity in higher concentrations of 50 and 100 µM (IC 50 = 59.3 µM).

Next, we investigated the capacity of the Dox-loaded folate-functionalized nanoparticle (MSN-LFA-Dox) to improve the cytotoxic effects towards MCF-7 cells after 24 h treatment (Figure 7B). For comparison, MCF-7 cells were treated with free Dox and Dox-loaded nonfunctionalized nanoparticles MSN-NH_2_-Dox and MSN-LFA-Dox, used in equimolar concentrations. As expected, free Dox showed concentration-dependent cytotoxicity (IC50 = 53.5 μM). Upon doxorubicin loading in MSN-NH_2_-Dox, a weak concentration-dependent drop in cell viability was observed. Nevertheless, it did not reach 50% of cell viability inhibition within the tested concentration range, and thus IC 50 was not determined. In contrast, functionalized doxorubicin-loaded MSN-LFA-Dox exerted the highest cytotoxicity (IC50 = 23.7 μM) with more pronounced effects at concentrations of 25 μM and higher.

Further, to test whether the observed increased cytotoxicity of MSN-LFA-Dox is due to the drug loading and not to a simple combination of MSN-LFA and free Dox, MCF-7 cells were incubated with a mixture of Dox + MSN-LFA nanoparticles (Table 3). The results showed that the drug-loaded MSN-LFA-Dox was more cytotoxic compared to the free combination of Dox + MSN-LFA.

#### 2.6.2. Phase Contrast Micrographs

**Figure 8:**
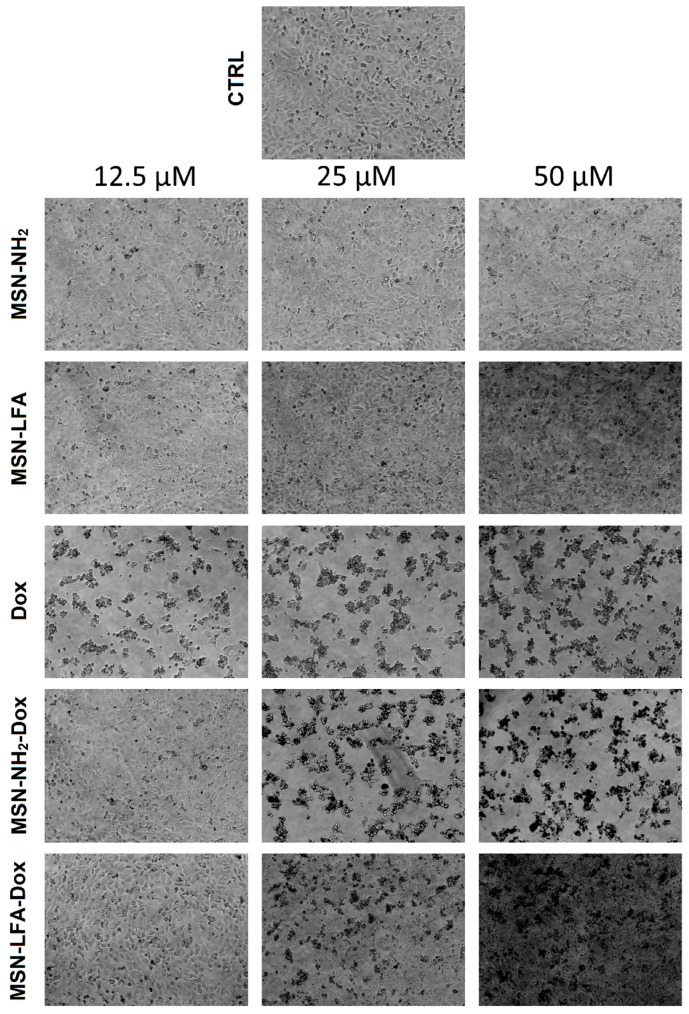
Phase contrast micrographs of MCF-7 cells, treated for 24 h with either non-loaded (MSN-NH_2_, MSN-LFA) or loaded (MSN-NH_2_-Dox, MSN-LFA-Dox) nanoparticles or Dox. All images are taken at 100× magnification.

In order to visually confirm the validity of the observations, as well as gain further insights into the modes of action of the test substances, we collected phase micrographs of treated and control MCF-7 cells immediately before the MTT assay (Figure 8). The cell morphology observations of the test samples were concordant with the MTT results. As seen, Dox treatment caused significant decreases in the number of viable cells. Moreover, it is visible that with doxorubicin-loaded MSN-NH_2_-Dox and MSN-LFA-Dox, there is a dark coloration of the cells, and this effect was concentration-dependent and most pronounced at the highest Dox concentration (50 μM).

#### 2.6.3. Cellular Uptake

**Figure 9:**
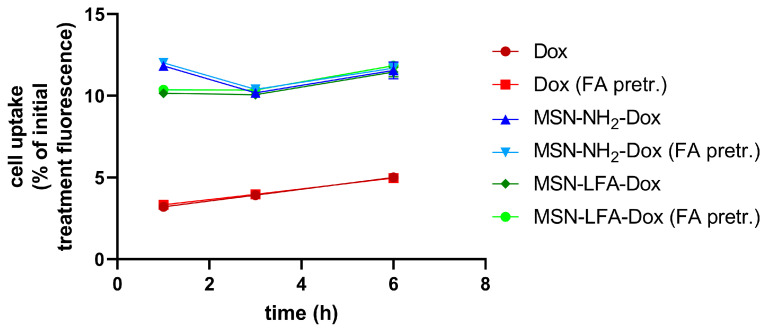
Cellular uptake, indicated by fluorescence intensities of MCF-7 cells, treated with Dox (red), MSN-NH_2_-Dox (blue), or MSN-LFA-Dox (green), normalized to fluorescence intensities in culture media at the initiation of treatment. Lighter red, blue, and green indicate that the cells were pretreated with FA-rich culture medium (FA pretr.) in order to induce FOL1R internalization and darker red, blue, and green indicate pretreatment with FA-poor culture medium, preserving active FOL1R.

We utilized doxorubicin’s fluorescent properties in order to evaluate the percentage of the drug inside the treatment culture media which reaches the cells. The possible effects of folic acid pretreatment on the ability of cells to uptake NPs was also monitored (Figure 9). After different time intervals (1 h, 3 h, and 6 h), we washed the cells and measured their fluorescence in a Synergy 2 plate reader. A linear trend of free Dox uptake with time was observed. The percentage of doxorubicin reaching the cells upon NP loading was several folds higher. With all NP types and with free Dox, there were no observed differences regarding folic acid pretreatment of MCF-7 cells.

#### 2.6.4. Fluorescence Micrographs

**Figure 10:**
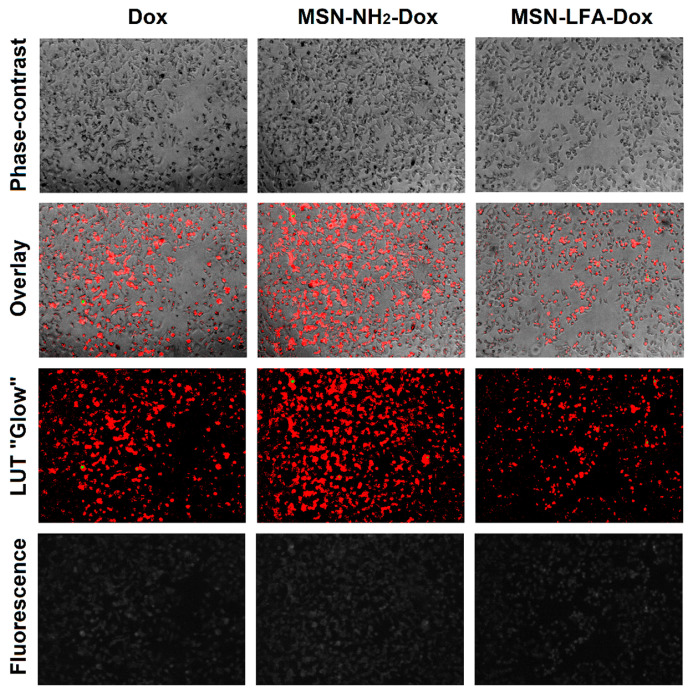
Fluorescence micrographs of MCF-7 cells, treated for 6 h with Dox, MSN-NH_2_-Dox, or MSN-LFA-Dox at 25 µM. The raw phase contrast images are presented for comparison (Raw), as well as raw fluorescence intensity (D fl.), and visually enhanced lookup table of fluorescence intensity (LUT), as well as an overlay between the LUT and phase contrast layers. Green fluorescence filter set with excitation 480–550 nm, dichroic mirror at 570 nm, emission ≥ 590 nm; longpass was used for fluorescence microscopy. All images are taken at 100 × magnification.

For visual confirmation of the cellular concentration of loaded and non-loaded Dox, we took fluorescent micrographs of the cells after 6 h treatment (Figure 10). Due to the low signal values, a lookup table (LUT) was used to make visualization and comparisons easier. The overlay of phase contrast images and the LUT of fluorescence images shows that all three groups of cells contain doxorubicin and the highest fluorescence is observed with MSN-NH_2_-Dox-treated cells.

#### 2.6.5. Cytotoxicity Evaluation on H9c2 Cardioblast Cell Line

**Figure 11:**
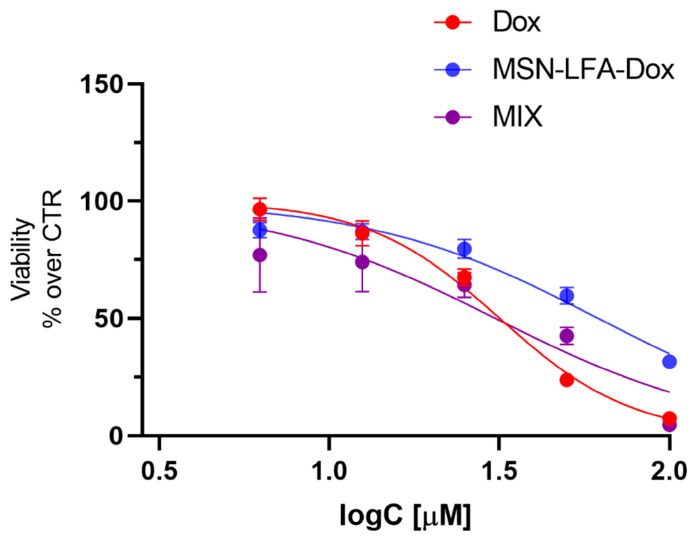
Cytotoxicity effects of free Dox (3.06–100 µM), physical mixture of Dox + MSN-LFA (MIX), and MSN-LFA-Dox on H9c2 cell viability after 24 h of treatment. The results are expressed as means ± SD of triplicate assays (*n* = 3). All groups were compared statistically vs. untreated controls by one-way ANOVA with Dunnet’s post-test.

One of the main restrictions in oncology practice is doxorubicin-related cardiotoxicity, which is a very common adverse drug reaction. Recently, nanosized drug delivery systems have been considered as a promising approach for reducing the Dox-induced toxicity [29,30,31]. Therefore, it is important to assess the potential in vitro cardiotoxicity of the Dox loaded in the functionalized nanoparticle (MSN-LFA-Dox) and to compare the effects with those of free Dox and the physical combination of Dox + MSN-LFA in H9c2 cardio blast cells (Figure 11). Free Dox (3.06–100 μM) caused concentration-dependent cytotoxicity (IC50 = 31.76 μM, 95% CI: 30.19–33.42 μM). When H9c2 cells were treated with the physical mixture of Dox + MSN-LFA, we obtained a similar result (IC50 = 30.87, 95% CI: 24.89 to 37.98 μM). However, the loading of Dox in the functionalized nanoparticle (MSN-LFA-Dox) showed significantly reduced cytotoxicity on cardioblasts (IC50 = 61.87, 95% CI: 56.59 to 68.12 μM) compared to free Dox and Dox + MSN-LFA.

## 3. Discussion

This study was focused on the preparation of a hybrid inorganic–lipid nanocarrier with a mesoporous silica nanoparticle core (MSN) which was further lipid-coated and reinforced with folic acid to improve the antiproliferative efficacy of doxorubicin to tumor cells. The performed in vitro tests showed that MSN-LFA-Dox enhance the cytotoxicity of doxorubicin in MCF-7 breast cancer cells while the doxorubicin-induced cardiotoxicity in H9c2 cardioblasts cells was attenuated.

The step by step preparation is schematically presented in Figure 1. The initial MSNs prepared in this work fall in the size range reported by previous work [32]. Our results show relatively small and uniform MSNs with negative zeta potential due to the predominance of free silanol surface groups. These findings coincide with previous reports [33,34]. The negative charge, however, is not sufficient to elucidate the complete dispersion in the aqueous medium and hence there are some aggregates identified in the micrometric range by the DLS analysis. It is established that zeta potential values above |30| mV are regarded as stable [35]. Upon amino-functionalization, as expected, the zeta potential changed to positive values (Table 1). This is a result of the introduction of amino groups on the surface of the MSNs. In double-distilled water (pH 7.2), they have the tendency to be protonated and positively charged. Despite the low value of the zeta potential, aggregates were not identified by the DLS data.

The next step in the proposed synthesis procedure included the coating with a lipid mixture. The chosen lipids included glyceryl dibehenate, glyceryl monostearate, and stearylamine. The glyceryl esters were selected as a result of their wide applicability as biocompatible lipid excipients in conventional and nanotechnological formulations [36,37]. Stearylamine is a primary amine utilized in the preparation of cationic liposomes. It possesses amphiphilic properties and could form lipid bilayers [38]. In the present work, we applied it both due to its amphiphilic properties and the availability of the primary amine group that could be further used for functionalization with folic acid. The lipid-coated empty nanoparticles without stearylamine had zeta potential of 5.82 ± 0.20 mV (preliminary experiments, see Supplementary Material Figure S1) while upon SA inclusion in the lipid coat the zeta potential increased to 50.30 ± 0.71 mV. A similar trend is observed in previous works with stearylamine [38,39]. This supports the claim for successful lipid coating onto the core MSN-NH_2_. The next step of FA coupling was also acknowledged by the demonstrated drop in the surface charge (Table 1).

In the case of the lipid-coated and folic acid-functionalized nanocarriers (MSN-LFAs), a significantly smaller size is observed (Table 1). It is rather associated with the presence of an additional small peak in the range of 10 nm. This is probably due to the preparation method and the presence of self-assembled lipid nanoparticles that do not layer on the inorganic core. In this case, the zeta potential does not reflect the expected physicochemical stability of the particles. The polymeric steric stabilization due to the poloxamer surface presence should be considered. The size and the particle surface charge are one of the leading parameters that contributes to nanotoxicity. In general, the small particles possess large surface areas, and thus they are reacting strongly with the biological components within the cell, leading to reactive species formation, lipid peroxidation, and cell injury. The performed preliminary toxicity evaluation of the empty nanoparticles showed that MSN-NH_2_ nanoparticles were nontoxic to MCF-7 cells even in the higher tested concentrations (up to 100 μM), while folate-coated MSN-LFAs showed an increased cytotoxicity in the concentrations over 50 μM.

The doxorubicin loading (MSN-NH_2_-Dox) is associated with a significant increase in the zeta potential value, and thus improved stability and narrower size distribution and PDI are observed (Table 1). This could be explained by the introduction of new surface amino groups to the nanocarrier due to the drug that are partially protonated (pKa 8.4 [40]) in double-distilled water (pH 7.2). We decided to apply aminated MSNs as core carriers because of the already existing data suggesting an improved doxorubicin loading in amino-functionalized nanoparticles as opposed to non-functionalized MSNs [41,42]. The most likely reason for the observed improvement is that doxorubicin’s amine groups are not protonated in the dispersion of the MSN-NH_2_ (pH ≈ 9.1). This explains the observed successful encapsulation and relatively high loading capacity (14.38 ± 1.53%). Similar assumptions were previously reported [41,43].

With respect to the encapsulation efficiency of the MSN-NH_2_-Dox, relatively low values are observed. However, the loading capacity is typical for the MSNs [41]. It should be noted that, in the present work, we did not aim at optimizing the encapsulation efficiency. Further studies are needed in order to provide the most favorable conditions thereof. The addition of lipid coat and FA functionalization increases the total weight of the nanocarrier and hence the loading capacity experiences a substantial drop. However, we consider these results as sufficient for the proof-of-concept study that can be further upgraded.

Some interesting observations were made with cytotoxicity studies in MCF-7 cell culture in vitro. Generally, the mechanisms of Dox cytotoxicity include DNA intercalation, topoisomerase II inhibition, and, as a secondary mechanism, anthracycline-induced reactive species formation, especially on the mitochondrial level [44]. Free Dox showed concentration-dependent cytotoxicity on MCF-7 cells, as expected. After MSN-NH_2_-Dox treatment, we noticed only a weak concentration-dependent drop in cell viability. It could be speculated that MSN-NH_2_-Dox nanoparticles may not readily release doxorubicin for the treatment period, and this results in attenuated cytotoxicity after 24 h treatment. The higher cytotoxicity of non-loaded folate-functionalized NPs (MSN-LFAs) compared to MSN-NH_2_-Dox is probably related to the higher NP load in the cells, as well as the potentially increased internalization. This statement is supported by phase contrast micrographs, showing that cells are darker after MSN-NH_2_-Dox treatment. A possible explanation for this observation is that there are strong electrostatic interactions between the negatively charged cell membranes and positively charged MSN-NH_2_-Dox NPs [45]. Fluorescence intensity measurements over time also showed initially higher fluorescence with MSN-NH_2_-Dox after one hour which also confirms the role of electrostatic interactions, although this method alone does not unequivocally show whether the NPs are internalized or attached to the cell surface.

The doxorubicin-loaded MSN-LFA-Dox are prepared based on the MSN-NH_2_-Dox which are characterized by better stability and narrow size distribution. This observation is retained and only a small increase in size comparing the non-coated drug-loaded nanoparticles is evident. Probably, doxorubicin affects the lipid layering and no very small particles are identified (Table 1). The release data demonstrated sustained and incomplete release within the tested 24 h period. The lipid coating, as expected, resulted in a decrease in the release rate as the drug diffusion is hindered by the hydrophobic layer on the nanoparticle’s surface. The results (Figure 6) showed higher release in the medium with pH 5.5 in comparison to the pH 7.4. Such a tendency was reported before [46]. A possible explanation for the pH-dependent release behavior is the more favorable doxorubicin desorption in acidic environments as already proposed in the literature [41,42]. The lipid coat served as a capping agent which additionally sustained the release in both pH media due to its lipophilic nature. This is achievable due to pre-surface grafting, i.e., the drug is loaded and then the nanocarrier surface is modified which is the most preferable strategy [47]. The observed behavior could be considered advantageous as the drug availability will be higher at the acidic conditions typical for the tumor microenvironment [28] in comparison to the healthy tissues [27].

An important observation from cell viability experiments was that Dox loading in folic acid-functionalized nanoparticles (MSN-LFA-Dox) showed the highest cytotoxicity on MCF-7 cells, compared to the effects of free Dox and MSN-NH_2_-Dox. To confirm that the observed effects are due to the drug loading, the cytotoxic effects of MSN-LFA-Dox were compared to the effects of the mixture of unloaded Dox + MSN-LFA. The results confirm the higher toxicity of the drug-loaded MSN-LFA-Dox toward MCF-7 cells. For cytotoxicity comparison, we rely on IC50 values, which are a robust parameter, highlighting the stronger cytotoxicity by MSN-LFA-Dox (IC50 = 23.7 μM). The observed effects could be partially attributed to the nanocarrier itself, especially in the highest tested concentrations of 50–100 μM. The transport of nanoparticles within the cells is related to the chemical binding between drug and surface derivatized nanoparticles. It is known that free Dox accumulates in the nucleus within the cell. The cell uptake experiments showed that the percentage of doxorubicin reaching the cells upon NP loading was several folds higher compared to free Dox. Fluorescence micrography and fluorescence intensity measurements showed that this was accompanied by internalization in MCF-7 cells. Most probably, the differences between the cytotoxicity effects of non-loaded and loaded Dox might be partially related to different internalization pathways. The observation of electrostatic effects introduces an additional variable influencing the cellular affinity, alongside FA functionalization, thereby complicating the interpretation of folic acid’s specific contribution. In addition, FA effects merely in in vitro models might not provide the whole picture of the potential improved cellular internalization and cytotoxicity [48].

Doxorubicin cardiotoxicity is a well known limitation for its clinical use. The toxic reactions are related to excess reactive oxygen species production (ROS) and mitochondrial dysfunction and DNA damage through topoisomerase 2β inhibition due to the oxidative stress mechanisms [49]. Dox loading in nanoparticulate systems is an approach for the achievement of targeted delivery and better antiproliferative efficacy toward tumor cells, but also to control and decrease the toxic effects of doxorubicin in healthy tissues. The study performed on H9c2 cells showed a significantly reduced cytotoxicity of MSN-LFA-Dox on cardioblasts, compared to Dox alone and the free combination of Dox + MSN-LFA.

In conclusion, this study proved that Dox loading in the hybrid inorganic–lipid nanocarrier MSN-LFA provides an opportunity to improve Dox’s antiproliferative efficacy in breast cancer MCF-7 cells and to decrease the drug-induced toxicity in H9c2 cells in vitro. Despite the well proven benefits of nanoscaled drug delivery systems, there are still issues that should be taken into account. To provide a clearer understanding of the observed effects of MSN-LFA-Dox and the role of folic acid for active targeting toward the tumor cells, future studies should aim to control for zeta potential in both functionalized and non-functionalized MSNs, and to incorporate cell lines known to lack FOLR1 expression as controls. Although these findings are encouraging, it is important to note that all conclusions are derived solely from in vitro experiments. This inherent limitation underscores the necessity for future in vivo studies to confirm the targeting specificity, biodistribution, pharmacokinetic behavior, and overall safety. Systematic in vivo validation will be essential to substantiate the translational relevance of our observations and to strengthen the overall robustness of the conclusions drawn here.

## 4. Materials and Methods

Cetyltrimethylammonium bromide (CTAB), triethanolamine (TEA), tetraethyl orthosilicate (TEOS), ethanol, anhydrous toluene, 3-Aminopropyltriethoxysilane (APTES) were purchased from Merck (Rahway, NJ, USA). Lutrol F127 was purchased from BASF (Ludwigshafen, Germany); N-ethyl-N′-(3-(dimethylamino)-propyl) carbodiimide (EDC), N-hydroxysuccinimide (NHS) were procured from Sigma Aldrich (Burlington, MA, USA); Folic acid (FA), and octadecylamine (stearylamine, SA) were obtained from ThermoFischer GmbH (Kandel, Germany). Chloroform was supplied by Fischer Chemical (Fischer Scientific, Loughborough, UK). Glyceryl dibehenate (GDB) as Compritol^®^ 888 ATO was kindly gifted by Gattefosse, Saint-Priest, France, and Glycerol monostearate type II (GS) as Imwitor 900 K was donated by IOI Oleo GmbH, Hamburg, Germany. All other reagents were of analytical grade.

### 4.1. Mesoporous Silica Nanoparticle Preparation and Amino-Functionalization (MSN-NH_2_)

MSNs with relatively small size were prepared using a previously published method [32] and underwent several modifications. In the procedure used, cetyltrimethylammonium bromide (CTAB) was used as a template surfactant, and the reaction was performed under basic conditions. For MSN synthesis, 0.5 g of CTAB and 0.2 g of triethanolamine (TEA) were dissolved in 20 mL of distilled water and maintained at a constant temperature of 40 °C. The mixture was further stirred at 650 rpm for 1 h, providing micelle formation. Then, 1.5 mL of tetraethyl orthosilicate (TEOS) was added slowly as fine droplets under continuous stirring, and the mixture was maintained under the same conditions for 12 h. MSNs were further collected by centrifugation at 15,000 rpm for 30 min and washed with ethanol to remove any residual reactants. The template was removed using the calcination method at 600 °C for 12 h (Muffle furnace SNOL 4/900, SnolTherm, UAB, Narkunai, Lithuana). MSNs were finally dried under vacuum for 24 h.

Amino modification of MSNs was performed using 3-aminopropyltriethoxysilane (APTES) as an agent for post-synthesis. In a typical procedure [50], 1 g of the dried MSNs was dispersed in 30 mL of anhydrous toluene under an inert nitrogen atmosphere to prevent hydrolysis. APTES in a quantity equivalent to approximately 10 mol% of the surface silanol groups was added under continuous stirring. The mixture was further refluxed at 60 °C for 24 h to ensure efficient covalent attachment of aminopropyl groups on the surface of MSNs. Amino-modified samples were collected using centrifugation (30 min, 15,000 rpm) and were washed with anhydrous toluene and ethanol to remove unbound silane species. MSNs were finally vacuum-dried for 6 h.

### 4.2. Lipid Coating of the MSN-NH_2_

The synthesized MSNs were subsequently surface coated with a lipid layer. The preparation was based on the method reported by Atlibature et al. [21] with adaptation to introduce the MSN-NH_2_ within the lipid nanoparticles. For this purpose, the lipids of choice (GDB 87.5 mg, GS 87.5 mg, and SA, 12.5 mg) were dissolved in 25 mL chloroform. The organic solvent was evaporated in rotary evaporator Büchi Rotavapor R-124 (Büchi Labortechnik AG, Flawil, Switzerland) at 60 °C, 150 rpm, for 2 h for complete solvent removal and thin film formation.

Aqueous solution of Pluronic F127 (2% *w*/*v*) was used as a rehydration polar phase. The MSN-NH_2_ was dispersed in this solution (50 mg in 3 mL) and it was then added to the lipid film and sonicated for 2 min at 60% amplitude (Bandelin SonuPuls HD3100, Bandelin Electronics, Berlin, Germany) followed by vortexing for 2 more minutes (Vortex Genius 3, IKA-Werke GmbH & Co.KG, Staufen, Germany).

### 4.3. Folic Acid Functionalization

In order to chemically attach folic acid as a targeting ligand to the hybrid nanoparticles, the EDC/NHS reaction was applied according to the established procedure [51,52]. Folic acid (12.5 mg) was dissolved in 3 mL phosphate-buffered saline (PBS) with pH 7.4 and incubated in the presence of EDC (5 mg) and NHS (7.5 mg) at room temperature under dark and electromagnetic stirring for 24 h. The PBS was freshly prepared by dissolving 0.8 g NaCl, 0.2 g KCl, and 0.144 g Na_2_HPO_4_ in double-distilled water. The pH was adjusted by adding a few drops of 0.1 M HCl.

Afterwards, the MSN-L (as dispersion) was added to this solution and stirred for another 24 h under dark. The unreacted chemicals were removed by dialysis (cellulose membrane, 1–5 kDa MWCO, Spectra/Por ^®^, Sigma Aldrich, (Burlington, MA, USA) against double-distilled water for 12 h.

### 4.4. Doxorubicin Loading and Encapsulation Efficiency

Doxorubicin (100 mg) was incorporated into mesoporous silica nanoparticles (MSN-NH_2_) via a solvent impregnation technique. The drug was initially dissolved in 10 mL of distilled water, after which 100 mg of MSN-NH_2_ was dispersed into the solution using sonication for 2 min to ensure uniform suspension. The resulting dispersion was subjected to continuous magnetic stirring at 600 rpm, under light-protected conditions, until the solvent had completely evaporated. The formed doxorubicin-loaded nanoparticles (MSN-NH_2_-Dox) were subsequently rinsed three times with ethanol and distilled in water to remove any unbound drug residues. Finally, the nanoparticles were dried in dark conditions at 3 °C. These nanoparticles were subsequently used for the preparation of the lipid coating and folic acid functionalization yielding the MSN-LFA-Dox samples.

In order to evaluate the encapsulation efficiency, the prepared MSN-NH_2_-Dox was washed with water and ethanol repeatedly and centrifuged (15,000 rpm for 20 min) between each rinse. The supernatants were collected and the amount of free drug was calculated based on the UV absorption at 480 nm. The encapsulation capacity (*EE*, %) and loading capacity (*LC*, %) were estimated based on the following equations:

(1) $$EE,\;\%=\frac{Total\;doxorubicin-Free\;doxorubicin\;in\;the\;supernatant}{Total\;doxorubicin\;added}$$

(2) $$LC,\;\%=\frac{Total\;doxorubicin-Free\;doxorubicin\;in\;the\;supernatant}{Total\;weight\;of\;the\;Dox-loaded\;nanoparticles}$$

### 4.5. Photon Correlation Analysis of Particle Size, Polydispersity Index (PDI), and Zeta Potential

Aqueous dispersions of the nanoparticles obtained at the different steps were subjected to dynamic light scattering analysis for particle size, polydispersity index, and surface charge evaluation. The dispersions were diluted with double-distilled water in 1:10 ratio prior to measurement. Three independent measurements were conducted at 25 °C and 173° scattering angle with the help of Zeta-master (Malvern Instruments, Worcestershire, UK).

### 4.6. Transmission Electron Microscopy (TEM)

Aqueous dispersions of each sample were diluted 1:10 with double-distilled water. A drop of each sample was placed on a copper grid and air dried. The morphology of the hybrid nanoparticles was visualized with JEOL JEM 2100 h STEM (JEOL, Tokyo, Japan) at 200 kV and with point resolution of 0.23 nm at different magnifications.

### 4.7. Nitrogen Physisorption

The textural characteristics of the synthesized materials were evaluated through nitrogen adsorption isotherms measured at −196 °C, using a Quantachrome Nova 1200e analyzer (Anton Paar, Boynton Beach, FL, USA). The Brunauer–Emmett–Teller (BET) method was employed to calculate the specific surface area, while the total pore volume was derived at a relative pressure (p/p_0_) of approximately 0.99. Pore size distribution and average pore diameter were assessed via the Non-Local Density Functional Theory (NLDFT) approach, and the micropore volume was determined using the t-plot (V-t) method.

### 4.8. X-Ray Powder Diffraction (PXRD)

X-ray powder diffraction patterns were collected with a constant step 0.02° 2θ in the range 5.3–80° 2θ on a Bruker D8 Advance diffractometer (Cu Kα radiation λ = 1.1518 Å, LynxEye detector). The low-angle part of the patterns was collected from 0.3° to 5° 2θ using a manually adjustable knife-edge anti-scatter screen attachment of the primary beam. Phase identification was performed with the Diffracplus EVA using ICDD-PDF2 Database (2024). Mean crystallite sizes were determined from the diffraction peaks broadening with the Topas-4.2, using the protocol for the size–strain tool implemented in the program.

### 4.9. Thermogravimetric Analysis (TGA)

Thermal analysis of the samples was conducted using a LABSYSEvo thermal analyzer (SETARAM, Caluire, France) operating in dynamic mode. The measurements were carried out under a continuous flow of high-purity Ar gas. The temperature was gradually increased from 25 °C to 800 °C, enabling the evaluation of thermal stability, material decomposition, and possible phase transitions across the thermal range.

### 4.10. In Vitro Dissolution Test

The in vitro release characteristics were investigated by the dialysis bag method (Spectra/Por^®^ 12–14 kDa regenerated cellulose, Spectrum Laboratories, Inc. Rancho Dominguez, CA, USA). Phosphate buffers with pH 7.4 and 5.5 were used as release media. Different samples (MSN-NH_2_-Dox, MSN-LFA-Dox) equivalent to approximately 9 mg of the drug were placed in a pre-soaked membrane. They were incubated in a 100 mL release medium at 37 ± 0.5 °C and 100 rpm horizontal agitation. Aliquots were withdrawn at predetermined time intervals and replaced with fresh medium. The amount of doxorubicin released was evaluated based on previously established calibration in the corresponding buffer at 480 nm (Thermo Scientific Evolution 300, Madison, WI, USA).

### 4.11. Cell Experiments

MCF-7 cells were obtained from ECACC (Salisbury, UK). Cells were cultured at 37 °C, 5% CO_2_ and maximum humidity in DMEM medium with high glucose, L-glutamine, 10% fetal bovine serum, and without folic acid. For experiments, 1 × 10^4^ cells were plated in 96-well plates and left overnight to attach. Rat cardio myoblast cell line H9c2 (ECACC, Salisbury, UK) was cultured in DMEM high glucose medium, supplemented with 10% (*v*/*v*) heat-inactivated FBS, 2 mM L-glutamine, and 1 mM sodium pyruvate.

#### 4.11.1. Cell Viability Assay

The MTT assay [53] was applied in order to evaluate changes in MCF-7 and H9c2 cells’ viability and cytotoxic effects. Cells were treated for 24 h with Dox or Dox-loaded NPs with Dox concentrations of 3.06, 6.125, 12.5, 25, 50, or 100 µM or with the equivalent concentrations of non-loaded NPs. After this period, the culture medium was aspirated and exchanged with fresh culture medium, containing 0.5 mg/mL MTT and incubated for 3 h at 37 °C, 5% CO_2_, and maximum humidity. Then, the MTT-containing medium was aspirated and DMSO was added to each well in order to dissolve the formazan, formed by live cells. Absorbance was measured at 570 nm with reference wavelength of 690 nm in a Synergy 2 plate reader (BioTek Instruments, Inc., Highland Park, Winooski, VT, USA). Viability was expressed as percentage over the mean values of the control group.

#### 4.11.2. Cell Uptake Assay

In order to evaluate the cell uptake of loaded or non-loaded Dox, we measured its fluorescence at different time intervals. Cells were treated with 25 µM Dox, loaded in NPs or non-loaded, and fluorescence was read in a Synergy 2 plate reader (BioTek Instruments, Inc., Highland Park, Winooski, VT, USA). The initial fluorescence inside culture medium with test substances was read immediately after treatment and after specific time intervals of 1, 3, and 6 h; a portion of treated wells was washed twice with PBS and fluorescence intensity was read at excitation 480 nm and emission 620 nm. Uptake values were expressed as percentage of fluorescence in cells, compared to initial fluorescence in the treatment medium.

#### 4.11.3. Phase Contrast and Fluorescence Micrography

Before the MTT treatment or after the fluorescence intensity readouts, cells were subjected to visual inspection via phase contrast or fluorescence microscopy. Images were taken at 100×magnification with a digital camera (Optikam Pro 8LT-4083.18LT) and mounted on an inverted Optika XDS-2 microscope in phase contrast mode or fluorescence mode with NFP-1 fluorescence power supply, mercury lamp, and a green fluorescence channel with 480–550 nm excitation filter, 570 nm dichroic filter, and 590 nm long pass emission filter. Images were analyzed on ImageJ software (version 1.54 p, National Institute of Health, Bethesda, MD, USA) [54] for flatfield correction with BaSiC plugin [55] and for enhanced visualization, “glow” lookup table was applied. For generating overlays between phase contrast and lookup table-edited images, they were aligned with DS4H Image Alignment plugin DS4H Image Alignment (DS4H-IA), an open-source ImageJ/Fiji plugin for aligning multimodality 2D microscopy images.

#### 4.11.4. Statistical Analysis

All statistical analyses were performed on GraphPadPrism software, version 8 (GraphPad Software, San Diego, CA, USA). Dose–response data were analyzed using nonlinear regression by least squares fitting a four-parameter logistic curve, including estimation of the IC_50_ values. To enable direct comparison across treatments, the top and bottom plateaus of the dose–response curves were constrained to fixed values of 100% and 0%, respectively, ensuring consistent normalization of maximal and minimal responses across datasets.

## 5. Conclusions

In this proof-of-concept study, we successfully synthesized folic acid-functionalized, lipid-coated mesoporous silica nanoparticles (MSNs) and demonstrated their potential as targeted drug delivery systems for doxorubicin in MCF-7 breast cancer cells. The hybrid nanoparticles displayed favorable physicochemical characteristics, including nanoscale size, sustained drug release—especially under acidic conditions typical of the tumor microenvironment—and enhanced stability following surface modification. In vitro assays revealed that doxorubicin-loaded, folate-functionalized nanoparticles achieved superior cytotoxic effects and higher cellular uptake compared to both non-functionalized and free drug controls. Notably, even non-loaded folate-functionalized nanoparticles exerted moderate cytotoxicity, suggesting possible intrinsic activity or increased internalization. Overall, the favorable physicochemical profile and in vitro cytotoxicity scores indicate that folic acid-functionalized hybrid nanoparticles are promising candidate chemotherapeutic agents to breast cancer cells. However, their targeting selectivity needs to be confirmed by generating stronger experimental proof. Further studies are warranted to optimize the formulation parameters and evaluate the in vivo therapeutic potential of these nanocarriers.

## Figures and Tables

**Table 1:** Particle size, PDI, and zeta potential of all prepared nanoparticles. The results represent the mean value of 3 independent measurements ± SD.

Sample Coding (Full Name)	Z-Average, nm	PDI ^1^	Peak 1, d_mean_ by Intensity, nm	Peak 1 Area, %	Peak 2, d_mean_ by Intensity, nm	Peak 2 Area, %	Zeta Potential, mV
MSN (mesoporous silica nanoparticle)	98.05 ± 1.34	0.257 ± 0.03	123.95 ± 5.30	96.85 ± 0.49	3939.50 ± 294	3.15 ± 0.49	−21.4 ± 0.0
MSN-NH_2_ (amine-functionalized MSN)	162.55 ± 25.53	0.323 ± 0.05	236.9 ± 10.51	100	-	-	10.8 ± 0.6
MSN-NH_2_-Dox (amine-functionalized MSN loaded with doxorubicin)	135.05 ± 0.91	0.184 ± 0.01	168.40 ± 2.26	100	-	0	51.5 ± 0.4
MSN-LFA (lipid-coated and folic acid-functionalized MSN-NH_2_)	84.4 ± 6.14	0.465 ± 0.04	174.90 ± 22.06	96.35 ± 1.77	9.96 ± 3.09	2.4	6.3 ± 6.3
MSN-LFA-Dox (lipid-coated and folic acid-functionalized MSN-NH_2_ loaded with doxorubicin)	171.3 ± 8.49	0.278 ± 0.06	224.40 ± 21.78	100	-	0	2.1 ± 0.2

^1^ PDI—polydispersity index.

**Table 2:** Texture parameters.

Sample	S_BET_ m^2^/g	V_total_ cm^3^/g	D_av_ nm	S_mi_ m^2^/g	S_ext_ m^2^/g	V_mi_ cm^3^/g
MSN	858	1.38	6.4	177	681	0.06
MSN-NH_2_	155	0.66	17	-	155	-
MSN-NH_2_-Dox	32	0.13	17	-	32	-
MSN-LFA	10	0.03	10		10	
MSN-LFA-Dox	15	0.04	8		15	

S_BET_—specific surface area; V_total_—total pore volume; D_av_—average pore diameter; S_mi_—microporous specific surface area; S_ext_—external specific surface area; V_mi_—micropore volume.

**Table 3:** IC50 values of free Dox, mixture of Dox + MSN-LFA, and MSN-LFA-Dox on MCF-7 cell line.

Treatment	IC50, μM	95% CI, μM
Dox	53.5	44.85–94.26
MSN-LFA-Dox	23.7	18.84–30.20
Dox + MSN-LFA	37.0	27.10–42.80

## Data Availability

The original contributions presented in this study are included in the article/Supplementary Material. Further inquiries can be directed to the corresponding authors.

## Supplementary Materials

The following supporting information can be downloaded at https://www.mdpi.com/article/10.3390/ijms27021092/s1.

## Abbreviations

The following abbreviations are used in this manuscript:

MSN	Mesoporous Silica Nanoparticle
NP	Nanoparticle
FA	Folic acid
DLS	Dynamic light scattering
PXRD	Powder X-ray diffraction
TGA	Thermogravimetric analysis
EPR	Enhanced permeation and retention
ATR	Active transport and retention
GRAS	Generally regarded as safe
TEM	Transmission electron microscopy
PSD	Pore size distribution
PDI	Particle size distribution
