# Supplementary material for: Synthesis of Folic Acid-Functionalized Hybrid Mesoporous Silica Nanoparticles and In Vitro Evaluation on MCF-7 Breast Cancer Cells
Source: Int J Mol Sci. 2026 Jan 22;27(2):1092. doi: 10.3390/ijms27021092 (PMC12841693; doi:10.3390/ijms27021092)
Supplement: Supplementary file 1 [file ijms-27-01092-s001.zip › ijms-4006384-supplementary.pdf]

Supplementary material

Synthesis of folic acid functionalized hybrid mesoporous silica nanoparticles and in vitro evaluation on MCF-7 breast cancer cells

Marta Slavkova [1*†], Yordan Yordanov [2†], Christina Voycheva [1], Teodora Popova [1], Ivanka Spassova [3], Daniela Ko- vacheva [3], Virginia Tzankova [2] and Borislav Tzankov [1*]

[1] Department of Pharmaceutical Technology and Biopharmaceutics, Faculty of Pharmacy, Medical University of Sofia, 1000 Sofia, Bulgaria; hvoycheva@pharmfac.mu-sofia.bg (C.V.); tpopova@pharmfac.mu-sofia.bg (T.P.)
[2] Department of Pharmacology, Pharmacotherapy and Toxicology, Faculty of Pharmacy, Medical University of Sofia, 1000 Sofia, Bulgaria; yyordanov@pharmfac.mu-sofia.bg (Y.Y.); vtzankova@pharmfac.mu-sofia.bg (V.T.)
[3] Institute of General and Inorganic Chemistry, Bulgarian Academy of Sciences, 1113 Sofia, Bulgaria; ispasova@svr.igic.bas.bg (I.S.); didka@svr.igic.bas.bg (D.K.)
* Correspondence: mslavkova@pharmfac.mu-sofia.bg (M.S.); btzankov@pharmfac.mu-sofia.bg (B.T.)
† These authors contributed equally to this work.

(a)

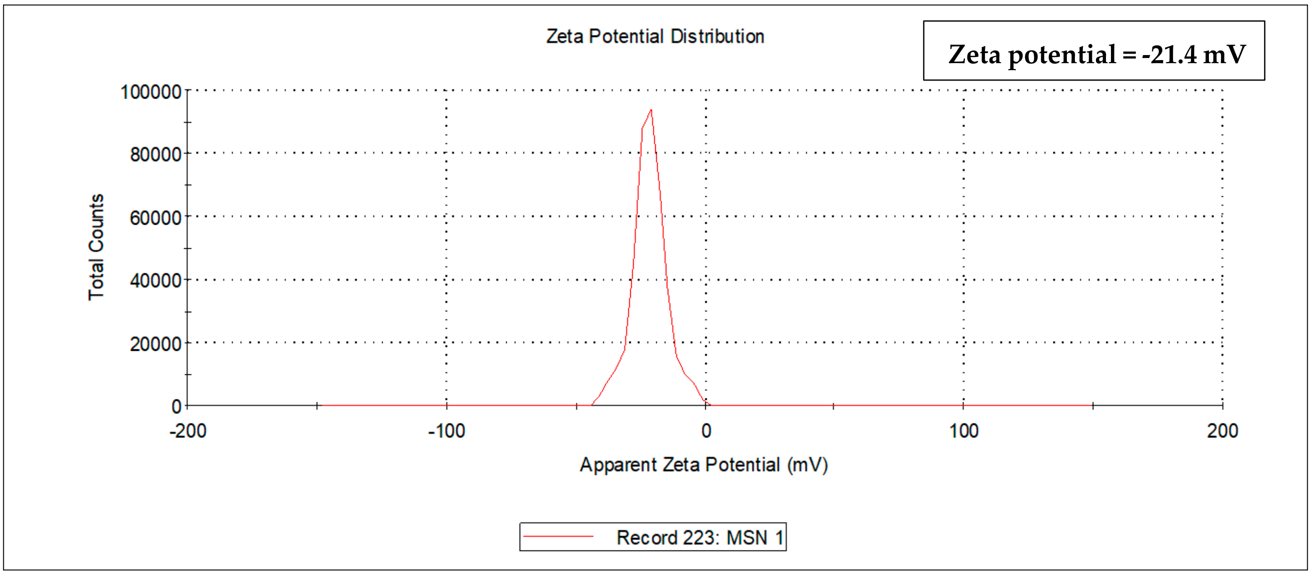

(b)

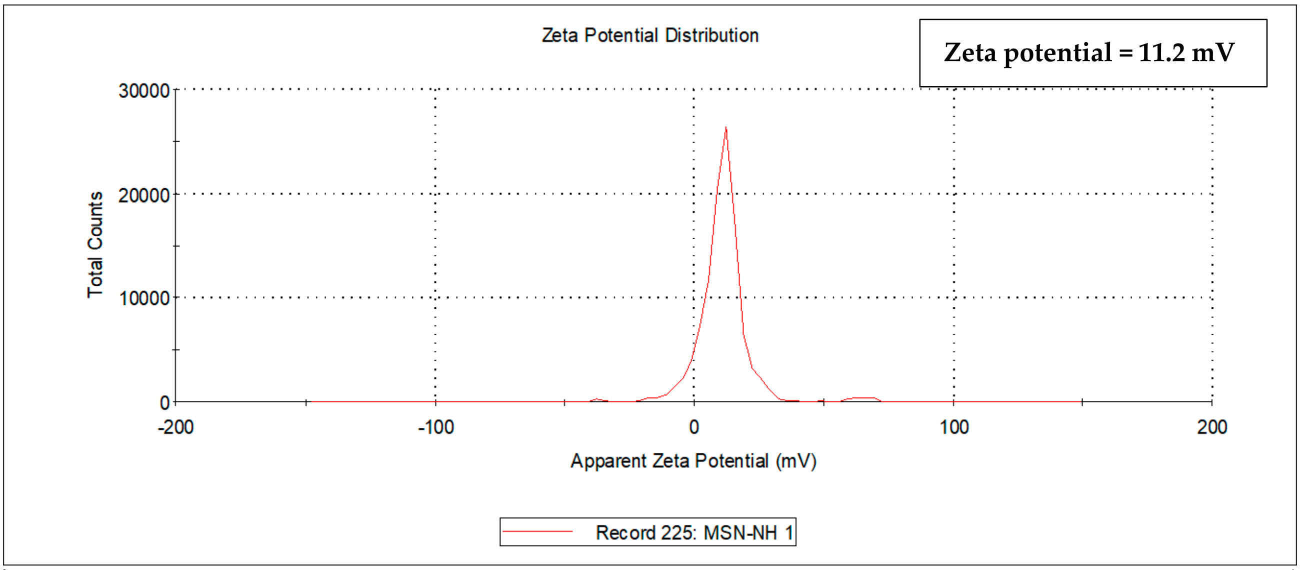

Zeta Potential Distribution
Zeta potential = 11.2 mV
Total Counts
30000
20000
10000
0
-200
-100
0
100
200
Apparent Zeta Potential (mV)
Record 225: MSN-NH 1

(c)

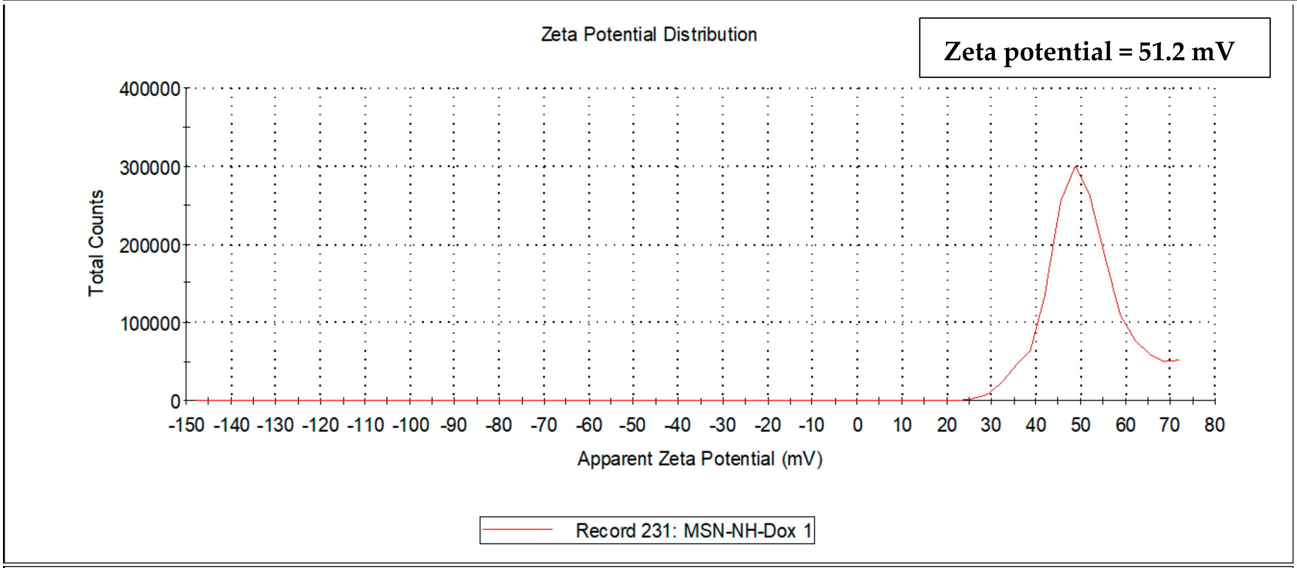

Zeta Potential Distribution
Zeta potential = 51.2 mV
Total Counts
400000
300000
200000
100000
0
-150 -140 -130 -120 -110 -100 -90 -80 -70 -60 -50 -40 -30 -20 -10 0 10 20 30 40 50 60 70 80
Apparent Zeta Potential (mV)
Record 231: MSN-NH-Dox 1

(d)

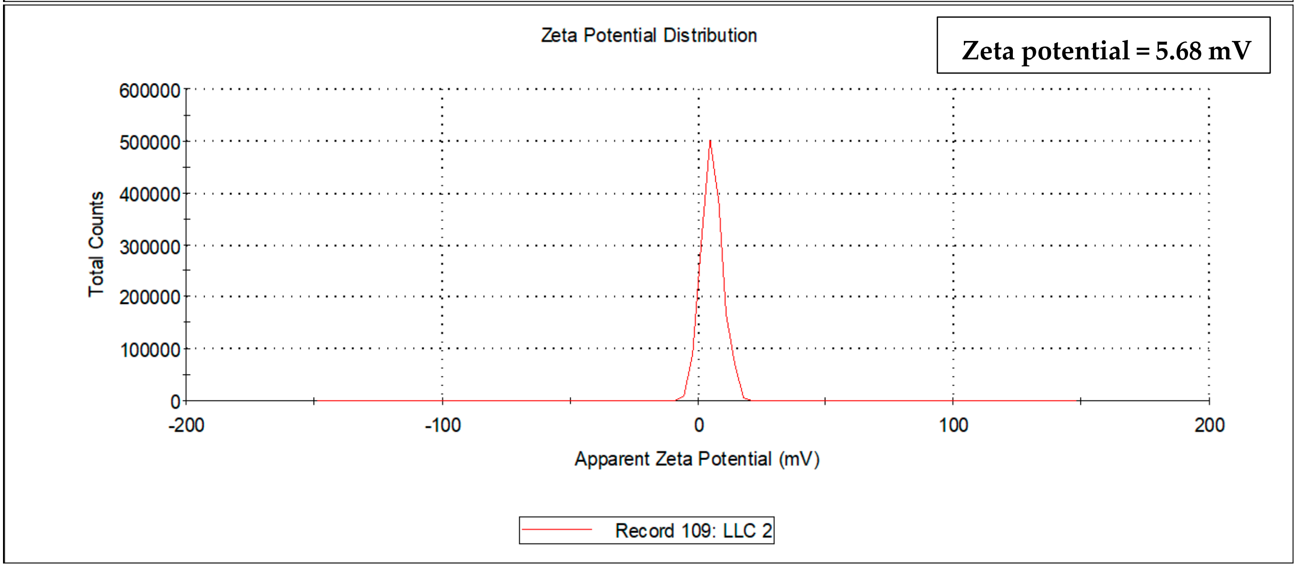

Zeta Potential Distribution
Zeta potential = 5.68 mV
Total Counts
600000
500000
400000
300000
200000
100000
0
-200
-100
0
100
200
Apparent Zeta Potential (mV)
Record 109: LLC 2

(e)

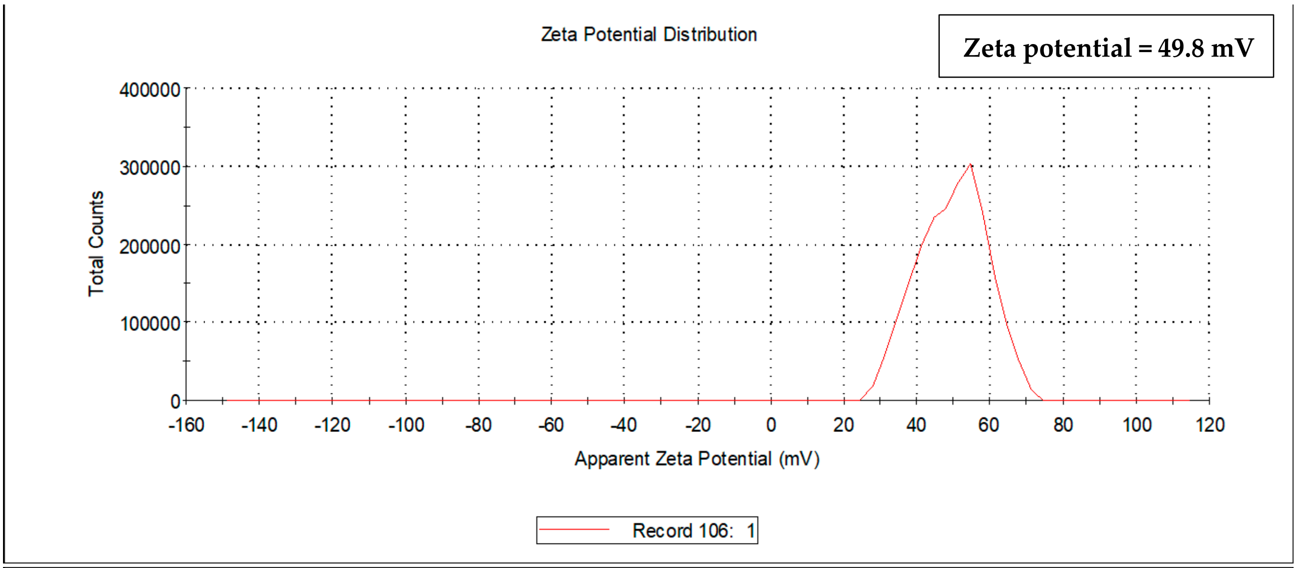

(f)

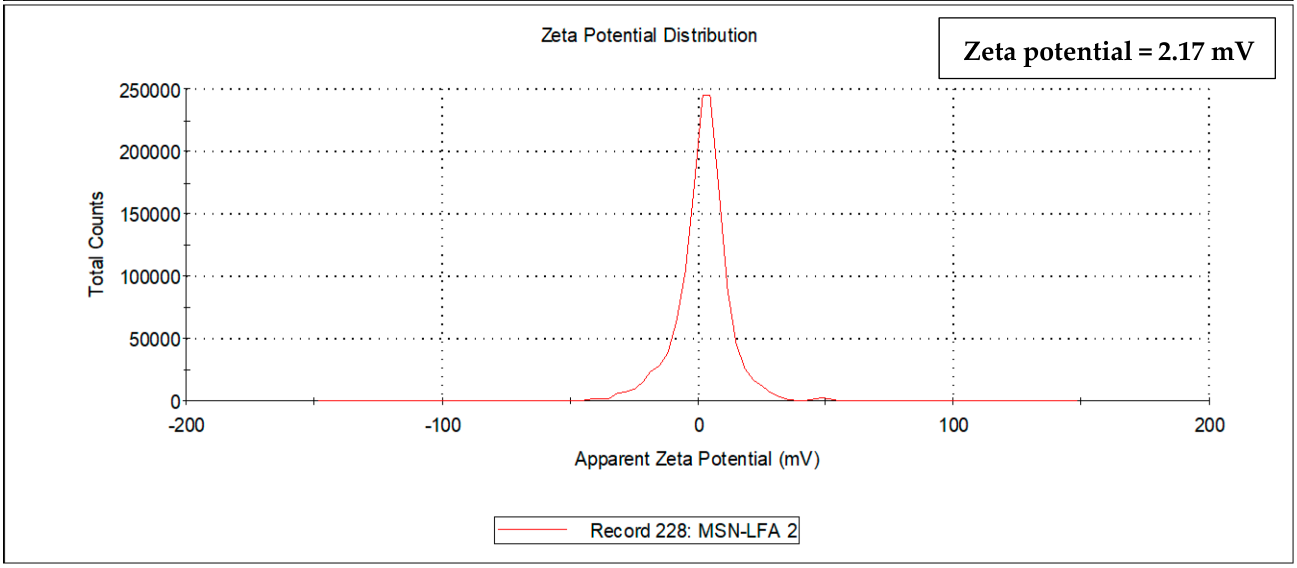

(g)

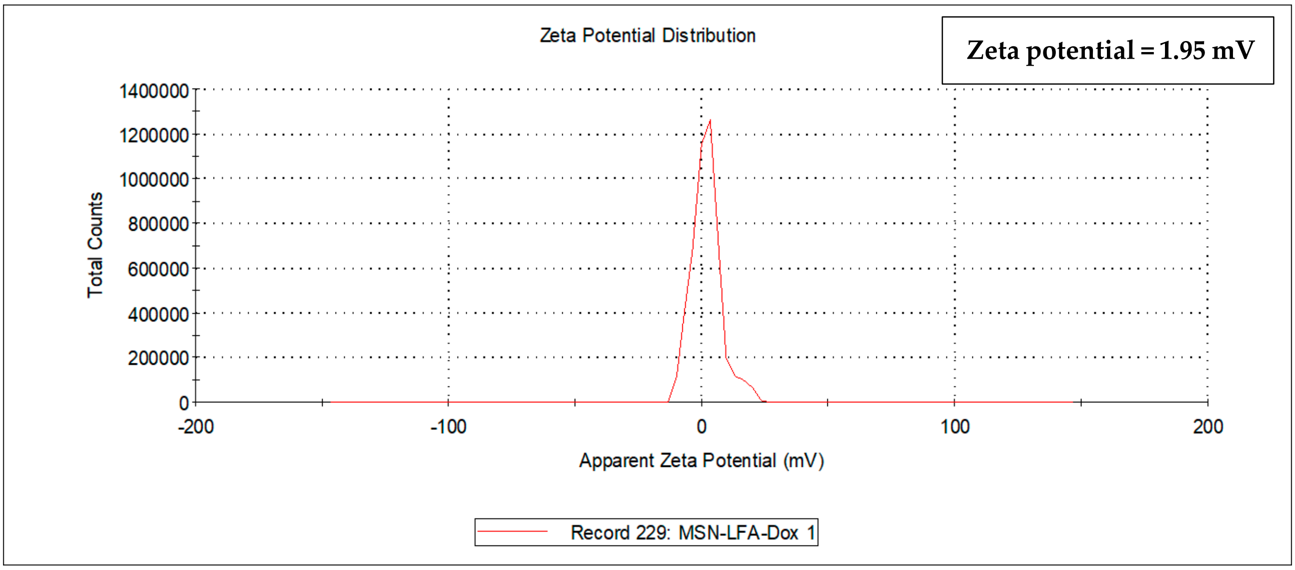

Figure S1. Representative zeta potential distribution graphs as generated by the Zeta-master for the following samples: (**a**) mesoporous silica nanoparticles (MSN); (**b**) amino-functionalized mesoporous silica nanoparticles (MSN-NH_2); (**c**) doxorubicin loaded amino-functionalized mesoporous silica nanoparticles (MSN-NH_2-Dox); (**d**) lipid coated MSN-NH_2 (without stearylamine in the lipid);

(**e**) lipid coated MSN-NH_2 (with stearylamine in the lipid); (**f**) lipid-coated MSN-NH_2 (with stearylamine in the lipid) functionalized with folic acid (MSN-LFA); (**g**) doxorubicin loaded MSN-LFA nanoparticles (MSN-LFA-Dox). All samples are measured after dilution 1:10 in double distilled water with pH=7.2, at 25°C and 173° scattering angle with disposable folded capillary cells DTS1070.
